# Evolving spike mutations in SARS-CoV-2 Omicron variants facilitate evasion from breakthrough infection-acquired antibodies

**DOI:** 10.1038/s41421-023-00584-6

**Published:** 2023-08-18

**Authors:** Shiqi Chen, Zehong Huang, Yue Guo, Huilin Guo, Lijuan Jian, Jin Xiao, Xiangyang Yao, Hai Yu, Tong Cheng, Yali Zhang, Ming Guan, Richeng Mao, Jiming Zhang, Ningshao Xia, Quan Yuan

**Affiliations:** 1 Department of Infectious Diseases, Shanghai Key Laboratory of Infectious Diseases and Biosafety Emergency Response, Shanghai Institute of Infectious Diseases and Biosecurity, National Medical Center for Infectious Diseases, Huashan Hospital, Fudan University, Shanghai, China; 2 State Key Laboratory of Vaccines for Infectious Diseases, Xiang An Biomedicine Laboratory, School of Public Health, Xiamen University, Xiamen, Fujian China; 3 National Institute of Diagnostics and Vaccine Development in Infectious Diseases, Xiamen University, Xiamen, Fujian China; 4 Department of Pulmonary Diseases, The First Affiliated Hospital of Xiamen University, Xiamen, China; 5 Department of Laboratory Medicine, Huashan Hospital, Shanghai Medical College, Fudan University, Shanghai, China

**Keywords:** Immunology, Molecular biology

Dear Editor,

SARS-CoV-2 inevitably evolves toward being more robust at multiplying. In the herd immunity era, mutations allowing SARS-CoV-2 to bypass the immunity acquired from vaccinations or infections may be essential in improving viral transmission fitness^1–5^. However, how evasion mutations drive the evolution remains to be elucidated.

Here, we characterized antigenic profiles of human convalescent plasmas (HCPs) from patients (*n* = 214) with different vaccination backgrounds and illness severities (Supplementary Table S1) in neutralizing pseudoviruses of multiple SARS-CoV-2 spike variants. Among unvaccinated populations, people with asymptomatic BA.2 infection or with mild to severe illness exhibited similar cross-variant antigenic profiles, which presented the highest neutralization antibody (nAb) titer against BA.2-related strains (BA.2 and BA.2.12.1), whereas were slightly reduced against BA.4/5 (~2–5×), moderately decreased against BA.1-related variants (~5–10×), and dramatically (> 10×) reduced in neutralizing early variants of D614G, Beta, and Delta (Supplementary Fig. S1a, upper panel). In contrast, the influence of variants on nAbs of HCPs from vaccinated people with BA.2 or BA.4/5 breakthrough infections appeared to be relatively slighter (Supplementary Fig. S1a, lower panel). Among tested variants, the Delta variant caused a 3.7–4.8× nAb reduction in patients with BA.2 breakthrough infections, and the BA.1-related viruses caused a 4.2–6.1× nAb attenuation in patients with BA.4/5 variant, whereas other variants affected little. Moreover, we measured the antibody titer of HCPs in binding spike proteins of D614G, BA.2, and BA.4/5, and the results were mainly consistent with those observed in neutralization tests (Supplementary Fig. S1b).

To compare antibody profiles between groups with various immunological statuses, we further established four age- and gender-matched subgroups (Supplementary Table S2), including breakthrough BA.2 infections following 2- (*n* = 18, 2d-BA.2-HCPs) or 3-dose (*n* = 17, 3d-BA.2-HCPs) of COVID-19 vaccine, breakthrough BA.4/5 infections following 3-dose of vaccine (*n* = 18, 3d-BA.4/5-HCPs), and unvaccinated BA.2 infections (*n* = 18, Unvaccinated BA.2-HCPs). As the 2d-BA.2-HCPs and 3d-BA.2-HCPs displayed remarkably similar antigenic profiles (Supplementary Fig. S2), we selected the 3d-BA.2-HCPs for further comparisons with the other two subgroups. In contrast to unvaccinated BA.2-HCPs, the 3d-BA.4/5-HCPs, and 3d-BA.2-HCPs showed significantly improved breadth in either neutralizing (Supplementary Fig. S3a) or binding (Supplementary Fig. S3b) with SARS-CoV-2 spike variants. However, neither BA.2 nor BA.4/5 breakthrough infections efficiently elicit nAbs against the XBB variant.

Looking back at the evolutionary trajectory of Omicron variants, introducing antigenic spike mutations on previous strains appeared to be an effective way to gain viral transmission fitness advantage for a new strain (Supplementary Fig. S4a, b). To elucidate the immune evasion role of amino-acid mutations during the BA.4/5 wave, we generated 34 variants with single amino-acid RBD mutations on the BA.4/5 spike backbone (Fig. 1a). The 3d-BA.4/5-HCPs and 3d-BA.2-HCPs were used for antigenic analyses of these variants (Supplementary Table S2). In contrast to that against the parent BA.4/5, the 3d-BA.4/5-HCPs showed significantly (*P* < 0.05) decreased nAb geometric mean titer (GMT) against the Y449N, K444R/N, V445P, and G446V/D by 1.9–4.7×, whereas the 3d-BA.2-HCPs presented 2.3–5.2× (*P* < 0.05) decreased nAb GMT against the D339H/Y, R346I/T, K444R/N, V445F/P, G446V/D, Y449N, N450K, N460S, A484V, and F490S/K/L (Fig. 1b). Weak correlation coefficients between nAbs against K444N, V445F, or G446V/D and others suggested that these mutations cause distinct antigenic signatures (Supplementary Fig. S5). Overall, for antibodies elicited by BA.2 or BA.4/5 breakthrough infections, mutations at aa 444, 445, 446, and 449 conferred a more marked neutralization evasion than other sites (Fig. 1c).

**Fig. 1:**
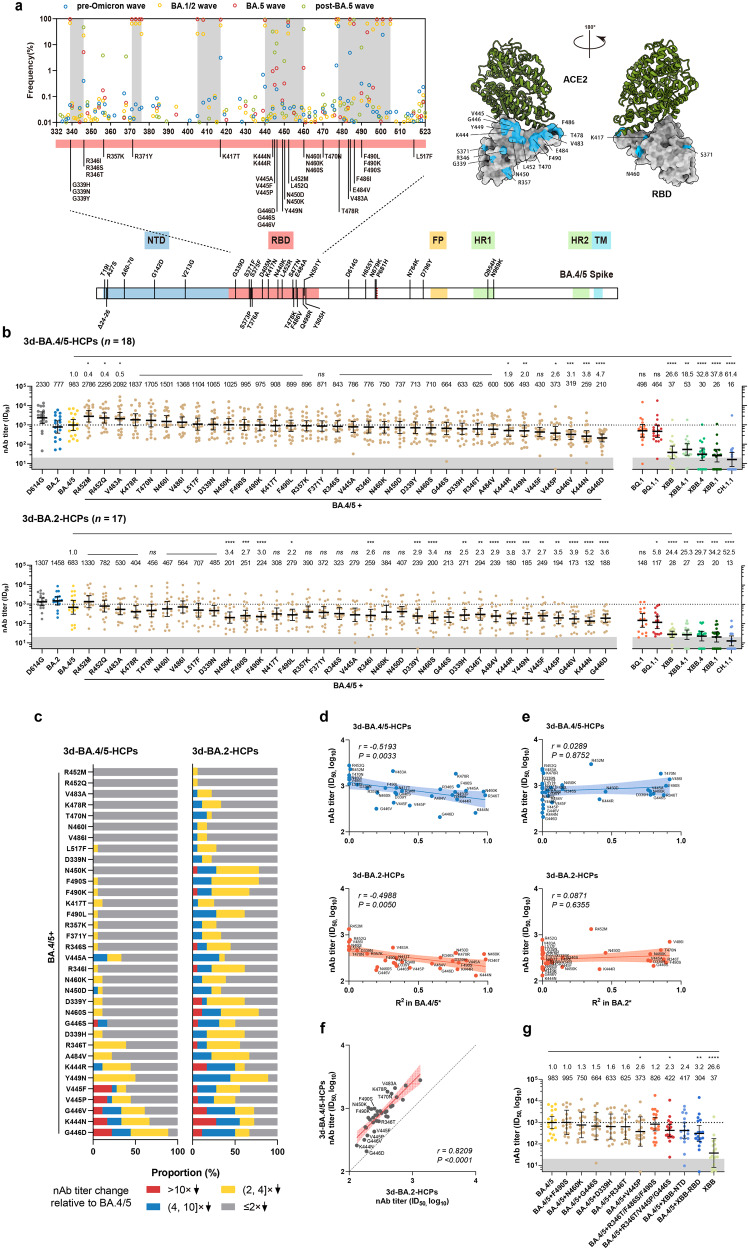
Characterizations of additional mutations in the context of BA.4/5 to enhance virus evasion from antibodies of breakthrough infections. **a** Frequencies of RBD mutations in different waves; 34 representative RBD mutations were selected for functional assessment. Schematics of involved mutations were presented in the BA.4/5 spike. L517 is not marked in the structure diagram as it is not on the RBD surface. NTD N-terminal domain, RBD receptor binding site, FP fusion peptide, HR heptad repeat, TM transmembrane. **b** Cross-neutralization profiles of the 3d-BA.4/5-HCPs (*n* = 18) and 3d-BA.2-HCPs (*n* = 17) to BA.4/5-RBD mutants (left panel) and newly emerged variants (right panel). Dark shadows indicate the limits of detections (ID_50_ = 20). The broken lines indicate the nAb GMT of HCPs against BA.4/5 for comparisons. **c** Proportions of HCPs according to the strata of nAb decrease against RBD mutants relative to BA.4/5. **d**, **e** Correlation of the nAb GMT of the breakthrough infection-derived HCPs against various BA.4/5-RBD mutants, with the *R*^2^ of exponential growth fit of the prevalence of the corresponding mutations in BA.4/5 (**d**) and BA.2 (**e**) strains. **f** Correlation of the nAb GMT titers in 3d-BA.4/5-HCPs and 3d-BA.2-HCPs against various BA.4/5-RBD mutants. In neutralization, assays of N417T, V445A, V445P, and G446S mutants, two samples in the 3d-BA.2-HCPs group, were not detected due to limited sample volume. **g** Neutralization profiles of the 3d-BA.4/5-HCPs against pseudoviruses bearing BA.4/5-XBB chimeric spikes. The antibody data against XBB, BA.4/5, and BA.4/5 bearing F490S, N460K, D339H, R346T, and V445P were the same as panel **b**. For panels **g** and **f**, data were plotted as the geometric mean with a 95% confidence interval. Mutations with sequence numbers < 10 from May to October 2022 were not fitted. In the correlation analysis of *R*^2^ with nAb titers, *R*^2^ for mutations with a decreasing trend (*k* < 0) in the time frame was assigned as 0. *P* values in panel **b** were calculated using two-tailed Wilcoxon signed-rank tests of paired samples; *P* values in panel **d**–**f** were the result of a two-tailed test for the Spearman rank correlation coefficient. *P < 0.05. **P < 0.01. ***P < 0.001. ****P < 0.0001. ns not significant, nAb neutralizing antibody, ID_50_ half-maximal inhibitory dilution.

Next, we analyzed the dynamic frequencies of viral sequences harboring these substitutions in viral sequences collected from May to December 2022 using the exponential growth model. The degree of conformity of changes in mutation prevalence to the exponential growth model, as determined by the *R*^2^ value, was used to measure the trend of mutation growth quantitatively (Supplementary Fig. S6). Notably, the *R*^2^ of all mutations in BA.4/5 descendants positively correlated with their escape capabilities (indicated by nAb GMT decreases) to either the 3d-BA.4/5-HCPs (*r* = −0.5193, *P* = 0.0033) or 3d-BA.2-HCPs (*r* = −0.4988, *P* = 0.0050) (Fig. 1d). In contrast, no significant correlation between nAb titer and *R*^2^ was observed in BA.2 and its descendants during the same period (Fig. 1e). For both 3d-BA.4/5-HCPs and 3d-BA.2-HCPs, mutations at aa 444, 445, and 446 caused more marked nAb attenuation than others (Fig. 1f). These findings revealed the statistical association between the mutation-related neutralization escape effect and their frequency growth in populations, suggesting that new variants with enhanced immune evasion appear to have greater fitness in spreading.

Several newly emerged variants in post BA.4/5 wave, such as BQ.1.1, XBB, and CH.1.1, also harbor RBD mutations at aa 346 and in the region of aa 444–446 (Supplementary Fig. S4). These variants exhibited striking escape to nAbs raised from BA.4/5 and BA.2 breakthrough infections (Fig. 1b). The BQ.1 and BQ.1.1 variants caused 4.6–5.8× nAb GMT reduction to the 3d-BA.2-HCPs compared to that against the BA.4/5, but have less impact on the 3d-BA.4/5-HCPs. Therefore, the competitive advantage of BQ.1/BQ.1.1 over BA.5 may be reflected in the ability to reinfect BA.2-infected people. Remarkably, the CH.1.1, XBB, and XBB-related sub-lineages (XBB.1, XBB.4, and XBB.4.1) led to the most striking nAb resistance, which caused an 18.5–61.4× nAb decrease for either 3d-BA.4/5-HCPs or 3d-BA.2-HCPs (Fig. 1b). Comparing with the BA.4/5, the CH.1.1 and XBB-related spikes present multiple NTD and RBD mutations but have the same S2 subunit (Supplementary Fig. S4b). To investigate how these mutations contribute to the neutralization escape, we generated four new spike variants, designated BA.4/5 + TM1 (BA.4/5 with R346T, F486S, and F490S), BA.4/5 + TM2 (BA.4/5 with R346T, V445P, and G446S), BA.4/5 + XBB-NTD (replace the NTD of BA.4/5 with that of XBB) and BA.4/5 + XBB-RBD (replace the RBD of BA.4/5 with that of XBB). Neutralization tests using the 3d-BA.4/5-HCPs (Fig. 1g) revealed that the latter three variants caused a 2.3–3.2× nAb decrease, whereas the BA.4/5 + TM1 did not show significant nAb reduction. This suggested that the additional V445P/G446S instead of the F486S/F490S contributed to the enhanced neutralization escape of XBB. Notably, the BA.4/5 + XBB-RBD with all RBD mutations of XBB (carrying R346T, L368I, V445P, G446S, F486S, and F490S) could only cause a 3.2× nAb decrease, which was roughly comparable to the alterations caused by the BA.4/5 + V445P and BA.4/5 + TM2 mutants. Compared to the NTD mutations, RBD mutations exhibited a relatively more considerable contribution to the neutralization escape of the XBB. However, it is noteworthy that neither NTD nor RBD mutations alone could enable such a prominent escape ability of the variant. Given that the BA.4/5 and XBB share an identical S2, these results demonstrated that both RBD- and NTD-targeting antibodies contribute significantly to spike-elicited neutralization activities and multiple mutations on the two domains have synergetic effects in causing remarkable evasion. A previous study demonstrated that mixtures of some RBD-targeting mAbs with NTD-targeting mAbs could lead to increased potency in neutralizing SARS-CoV-2, acting synergistically^6^. The XBB-NTD mutations may not only reduce the effectiveness of NTD antibodies but also eliminate their synergistic effects in facilitating the neutralization activities of RBD-antibodies, specifically those RBD antibodies that remain resistant to XBB-RBD mutations, and vice versa.

Furthermore, we tested four RBD-targeting mAbs (LY-CoV1404, COV2-2130, BD55-5840, and 85F7) that efficiently (IC_50_ < 0.2 μg/mL) neutralize the ancestral D614G, BA.2 and BA.4/5 (Supplementary Figs. S7a, b and S8)^3,7–9^. As shown in Supplementary Fig. S7a, the activity of LY-CoV1404 was markedly attenuated (> 10× reduction) by V445A/F/P, K444N, and G446D/V. For the COV2-130, five mutations (K444N, V445A/F/P, and G446D) that caused > 10× neutralization potency decrease. BD55-5840 was highly sensitive (> 10× reduction) to the mutations of D339H/N/Y, R346S/I/T, K444N, V445P, G446D and N450D. For the 85F7, the R346S/T, K444N/R, V445P, G446D/S/V and N450D exhibited strongly evasion (> 10× reduction). Notably, all mAbs almost completely lost their neutralization activities against BQ.1/BQ.1.1 and XBB variants. According to their recognizing modes, these mAbs all belong to class 3 mAb, and the escape mutations are mainly located at the RBD/antibody interfaces (Supplementary Fig. S7c). As a neutralization control and also an indicator for spike/ACE2 binding capabilities, recombinant human ACE2 protein (rhuACE2) displayed comparable potencies to most mutants (Supplementary Fig. S7a, left column). The effect of mutations on spike/ACE2 binding capacity does not seem to correlate with the evolutionary trend of both BA.2 and BA.4/5 subvariants (Supplementary Fig. S7d).

For SARS-CoV-2, the early VOC/VOI variants circulated from 2020 to 2021, such as Alpha, Beta, and Delta, appear to have enhanced transmissibility and extended cross-species tropism instead of striking immune escape^10^. However, under the herd immunity scenario, antigenic escape may have been the most favorable effect on viral transmission fitness and therefore became the primary factor in determining viral evolution direction. This hypothesis was partially evidenced by our findings that the frequencies of mutations with high escape potentials in BA.4/5 lineages exhibit higher growth momentums, suggesting that new variants with extensive immune evasion appear to have greater fitness in spreading. Following these results, it could be predicted that initial immunity conferred by current vaccines and/or natural infections may not protect against the challenge of mutated viruses in the following wave. And future escape mutations targeting individuals infected with strains such as XBB could be the main direction of the next round of virus evolution.

In summary, our study systematically characterized cross-neutralization profiles of antibodies acquired from breakthrough infections of BA.2 and BA.4/5 and revealed the predominant role of amino-acid substitutions at aa 346 and aa 444–446 in facilitating immune evasion. Moreover, the findings of the underlying association between mutation-related neutralization evasion and their prevalence growth suggest that new variants with enhanced immune evasion appear to have greater transmission fitness. These results highlight that we should keep up to strengthen the monitoring of SARS-CoV-2 evolutions and mutations and design broad-spectrum vaccines or drugs to achieve more effective COVID-19 control.

### Supplementary information

Supplementary Information

## Data Availability

The data that support this study are available from the corresponding authors upon request. All reagents, including antibodies, proteins, plasmids, and viruses, will be available upon request after a Materials Transfer Agreement is completed for non-commercial usage. Data used in prevalence dynamics analysis is available from the GISAID (https://www.gisaid.org) and covSPECTRUM (https://cov-spectrum.org).
