# Supplementary material for: Evolving spike mutations in SARS-CoV-2 Omicron variants facilitate evasion from breakthrough infection-acquired antibodies
Source: Cell Discov. 2023 Aug 18;9:86. doi: 10.1038/s41421-023-00584-6 (PMC10439137; doi:10.1038/s41421-023-00584-6)
Supplement: Supplementary file 1 — Supplementary Information [file 41421_2023_584_MOESM1_ESM.pdf]

Supplementary information for

Evolving spike mutations in SARS-CoV-2 Omicron variants facilitate evasion from breakthrough infection-acquired antibodies

Shiqi Chen[1,6], Zehong Huang[2,3,6], Yue Guo[1,6], Huilin Guo[2,3,6], Lijuan Jian[4,6], Jin Xiao[2,3], Xiangyang Yao[4], Hai Yu[2,3], Tong Cheng[2,3], Yali Zhang[2,3,*], Ming Guan[5,*], Richeng Mao[1,*], Jiming Zhang[1,*], Ningshao Xia [2,3,*] and Quan Yuan[2,3,*]

[1] Department of Infectious Diseases, Shanghai Key Laboratory of Infectious Diseases and Biosafety Emergency Response, Shanghai Institute of Infectious Diseases and Biosecurity, National Medical Center for Infectious Diseases, Huashan Hospital, Fudan University, Shanghai, China

[2] State Key Laboratory of Vaccines for Infectious Diseases, Xiang An Biomedicine Laboratory, School of Public Health, Xiamen University, Xiamen, Fujian, China

[3] National Institute of Diagnostics and Vaccine Development in Infectious Diseases, Xiamen University, Xiamen, Fujian, China

[4] Department of pulmonary diseases, The First Affiliated Hospital of Xiamen University, Xiamen, China

[5] Department of Laboratory Medicine, Huashan Hospital, Shanghai Medical College, Fudan University, Shanghai, China.

[6] These authors contributed equally to this work and shared the first authorship.

[*] **Corresponding authors:**

Yali Zhang (zhangyali@xmu.edu.cn); Ming Guan (guanming88@yahoo.com);

Richeng Mao (njxiaomao@163.com); Jiming Zhang (jmzhang@fudan.edu.cn);

Ningshao Xia (nsxia@xmu.edu.cn); Quan Yuan (yuanquan@xmu.edu.cn)

This file includes:

Materials and Methods

Supplementary References

Supplementary Figures S1 to S8

Supplementary Tables S1 to S2

Materials and methods

Human COVID-19 convalescent plasma samples

A total of 214 COVID-19 patients admitted to Huashan Hospital Affiliated of Fudan University (BA.2 wave, between March and June 2022) and the First Affiliated Hospital of Xiamen University (BA.4/5 wave, between July and August 2022) were enrolled for the study. Demographic characteristics, COVID-19 vaccination information, and clinical illness symptoms of all enrolled were extracted from the medical records obtained from the hospital where the patients received treatment after diagnosis. Plasma samples were isolated from centrifuged blood and then stored at -80 ℃ until used. The study was conducted according to the guidelines of the Declaration of Helsinki and approved by the Institutional Review Board of the Ethics Committee of Huashan Hospital (2022-721). Written informed consent was obtained for each participant.

Cell lines

ExpiCHO-S Cells (Thermo Fisher Cat# A29133) were cultured in the ExpiCHO Expression System (Thermo Fisher Cat# A29133) at 37℃ with 8% CO_2 on an orbital shaker platform. H1299 cells (cat# CRL-5803) were from ATCC and cultured in 10% Fetal Bovine Serum

(GIBCO cat# 10099141) supplemented Dulbecco' 's Modified Eagle Medium (DMEM, GIBCO cat# 11965092) at 37℃, 5% CO_2.

Monoclonal antibodies

Monoclonal antibodies tested in this study were constructed and produced at Xiamen University. For each antibody, variable genes were codon optimized for human cell expression and synthesized by General Biol (Anhui, China) into plasmids (EIRBdMie) that dual-promoter vector containing constant regions of human IgG1 heavy and light chains. Antibodies were expressed in ExpiCHO^{TM} Expression Medium (ThermoFisher, A2910002) by co-transfection of heavy and light chain expressing plasmids using ExpiFectamine^{TM} CHO Transfection Kit (ThermoFisher, A29129) and cells were cultured at 37℃ with shaking at 125 RPM and 8% CO_2. On day 5, antibodies were purified using MabSelectTM PrismA (Cytiva, 17549801) affinity chromatography.

Construction and production of variant pseudoviruses

Lentiviral-based pseudotyping particle (LVpp) bearing SARS-CoV-2 spikes were produced as previously described [1]. Plasmids containing SARS-CoV-2 spike variant-expressing cassettes, including that of Beta/B.1.351 (referring EPI_ISL_700428), Delta/B.1.617.2 (referring EPI_ISL_2723562), BA.1 (referring EPI_ISL_8324808), BA.1.1 (referring EPI_ISL_9640036), BA.2 (referring EPI_ISL_8253179), BA.2.12.1 (referring EPI_ISL_11704386), BA.4/5 (referring EPI_ISL_11550739), BQ.1 (referring EPI_ISL_15773424), BQ.1.1 (referring EPI_ISL_15514723), XBB (referring EPI_ISL_14917728), XBB.1 (referring EPI_ISL_15312882), XBB.4 (referring

EPI_ISL_15430349), XBB.4.1 (referring EPI_ISL_15602726), and CH.1.1 (referring EPI_ISL_15345176) were generated by site-directed site-specific mutagenesis on a previously described vector (EIRBsMie-dSwtG, containing codon-optimized spike gene from MN908947.3 with D614G substitution) [2]. The 18aa from the C-terminus of the spike was replaced with a HiBit bioluminescent tag (14aa, GSGVSGWRLFKKIS) [3]. HEK293T-F17 cells were grown to appropriate density before co-transfecting the spike-expressing plasmids, the packing plasmid of psPAX2, and the mNeonGreen reporter vector (pLVEF1αmNG) using Lipofectamine® 3000 (Thermo Scientific, L3000015). The supernatants were collected at 48 or 72 hours after transfection, filtrated by a 0.45-μm pore size filter, and were subsequently subjected to determine the titers in infecting huACE2-H1299 cells. Aliquot viral stocks were stored at -80°C freezer until use.

Pseudovirus neutralization assays

Neutralization assays were performed by incubating pseudoviruses with serial dilutions of monoclonal antibodies or plasmas and scored by the reduction in cellular fluorescent images. In brief, serially-diluted samples (3-fold series dilutions, from 1:30 to 1:21,870, 1:10 dilution will be supplemented when 1:30 dilution does not show significant inhibitory activity) were pre-incubated with the pseudovirus inoculum (1,800 GFU/well) for 1 hour. The mixtures were further incubated with the huACE2-H1299 cells pre-seeded in 96-well cell culture plates at 37°C in a CO_2 incubator. After a 2-day culture, cellular fluorescent images were acquired by using Opera Phenix's high-content imaging system (PerkinElmer). For each well, the total (H2B-mRuby3-activated) and LVpp-infected (mNeonGreen-activated) cell numbers were determined by the Columbus Image analysis

system (PerkinElmer). After normalization with the total cell number, the infection inhibition ratio of each sample at different dilutions was calculated by comparing it with the LVpp-only control. IC_{50} was defined as the dilution at which the relative light units were reduced by 50% compared with the virus control wells (virus + cells) after subtraction of the background in the control groups with cells only. The IC_{50} values were calculated using nonlinear regression in GraphPad Prism (version 9.3.1). An $ID_{50} \geq 20$ was defined as the cutoff value to determine SARS-CoV-2 nAb seropositivity. And an $ID_{50} < 5$ was assigned a value of 5. The performance of the neutralization assay used in our study has been well documented in our previous study [1].

Recombinant Protein production

Expression plasmids of recombinant spike proteins were constructed encoding for human codon-optimized sequences from wild-type SARS-CoV-2 (MN908947), BA.2 (EPI_ISL_8253179), and BA.4/5 (EPI_ISL_11542465). The ectodomain of these spike fragments (aa 1-1207, furin cleavage site mutated and HexaPro stabilized, fusing with a T4 trimeric motif) of Omicron were customs synthesized by General Biol (Anhui, China) and cloned into EIRBsMie vector, respectively, as previously described [4]. Expression constructs (StriFKHP, StriFK364HP, and StriFKA75HP) were verified by Sanger sequencing after plasmid isolation using the QIAGEN Miniprep kit (QIAGEN). Plasmids encoding proteins were transiently expressed in ExpiCHO cells and purified with a 5 mL Ni sepharose 6 Fast Flow column (Cytiva). Harvested and concentrated proteins are coated in polystyrene or chemiluminescent microplates for subsequent detection.

Anti-spike IgG measurements

Microplates pre-coated with recombinant spike proteins were as previously described [2]. For detections, serially-diluted samples (5-fold series dilutions, from 1:10 to 1:6250, 100 μl per well) were added to the wells, and the plates were incubated at 37°C for 30 min, followed by washing with PBST buffer [20 mM PBS (pH7.4), 150 mM NaCl, and 0.05% Tween-20]. Then, anti-human IgG (Thermo Fisher Scientific) for 30-min incubation, and then washing with PBST buffer again. Tetramethylbenzidine (Wantai) chromogen solution (100 μl per well) was added to each well. Ten minutes later, the chromogen reaction was stopped by adding 50 μl of 2 M H_2SO_4, and optical density (OD) 450 nm–630 nm was measured. The IgG titer was defined as the dilution limit to achieve a positive result (greater than the mean plus 3 SDs of ODs of negative controls). Each plate contained three tests of negative control plasmas, and their ODs were used to determine the cutoff value. Representative data from technical replicates were performed at least twice for plotting.

Prevalence data of subvariants and mutations

The current snapshot of Omicron subvariants data was taken from the EpiCoV database of GISAID (https://www.epicov.org) [5]. Variants were designated according to their Pango dynamic lineage classification [6]. Minor sublineages of each subvariant were grouped with their parental variant. The prevalence of point mutations in all SARS-CoV-2 sequences was downloaded from the file "spikeprot" of GISAID. The prototype Spike sequence (hCoV-19/Wuhan/WIV04/2019) was used as the reference. The prevalence of point mutations in BA.4/5 and its sublines was extracted from the data collated by covSPECTRUM

(https://cov-spectrum.org) [7] based on GISAID [5]. In the Settings options, all samples were selected, and the location is defined as the world.

Homology modeling

In this study, based on the reported complex structures of antibodies LY-CoV1404, 85F7, BD55-5840, and COV2-2130 with SARS-CoV-2, we obtained the complex structure of antibodies with BA.4 variant by homologous modeling. Homology modeling is performed by the Build Homology Model module in Discovery Studio 2017 R2 (DS) with default parameters. The program will calculate the DOPE score to evaluate the model quality and output the top 20 structures, and the structure with the highest ranking was chosen.

Statistical analysis

Propensity score matching is utilized for matching between different groups. And the age, gender, co-existing medical conditions, illness severity, and sampling days are employed for matching. When there are significant differences in diagnoses and sampling days between groups, priority is given to ensuring matching the first three characteristics. The Friedman test with Dunn's correction was applied to analyze differences among groups. The Spearman rank correlation coefficient was used for linear correlation analysis between the antibody titers and titers and the R^2. The prevalence of mutations was fitted by using the exponential growth model. Statistical differences were considered to be significant for two-tailed P values of < 0.05. Statistical analyses were conducted by GraphPad Prism (version 9.3.1) or R software (version 4.1.2).

Supplementary References

1 Chang, L. *et al.* The prevalence of antibodies to SARS-CoV-2 among blood donors in China. *Nat Commun* **12**, 1383, doi:10.1038/s41467-021-21503-x (2021).

2 Zhang, Y. *et al.* Cross-species tropism and antigenic landscapes of circulating SARS-CoV-2 variants. *Cell Rep* **38**, 110558, doi:10.1016/j.celrep.2022.110558 (2022).

3 Dixon, A. S. *et al.* NanoLuc Complementation Reporter Optimized for Accurate Measurement of Protein Interactions in Cells. *ACS Chem Biol* **11**, 400-408, doi:10.1021/acschembio.5b00753 (2016).

4 Wu, Y. *et al.* A recombinant spike protein subunit vaccine confers protective immunity against SARS-CoV-2 infection and transmission in hamsters. *Science translational medicine* **13**, doi:10.1126/scitranslmed.abg1143 (2021).

5 Shu, Y. & McCauley, J. GISAID: Global initiative on sharing all influenza data - from vision to reality. *Euro Surveill* **22**, doi:10.2807/1560-7917.Es.2017.22.13.30494 (2017).

6 O'Toole, Á. *et al.* Assignment of epidemiological lineages in an emerging pandemic using the pangolin tool. *Virus Evolution* **7**, doi:10.1093/ve/veab064 (2021).

7 Chen, C. *et al.* CoV-Spectrum: analysis of globally shared SARS-CoV-2 data to identify and characterize new variants. *Bioinformatics* **38**, 1735-1737, doi:10.1093/bioinformatics/btab856 (2022).

Supplementary Figures

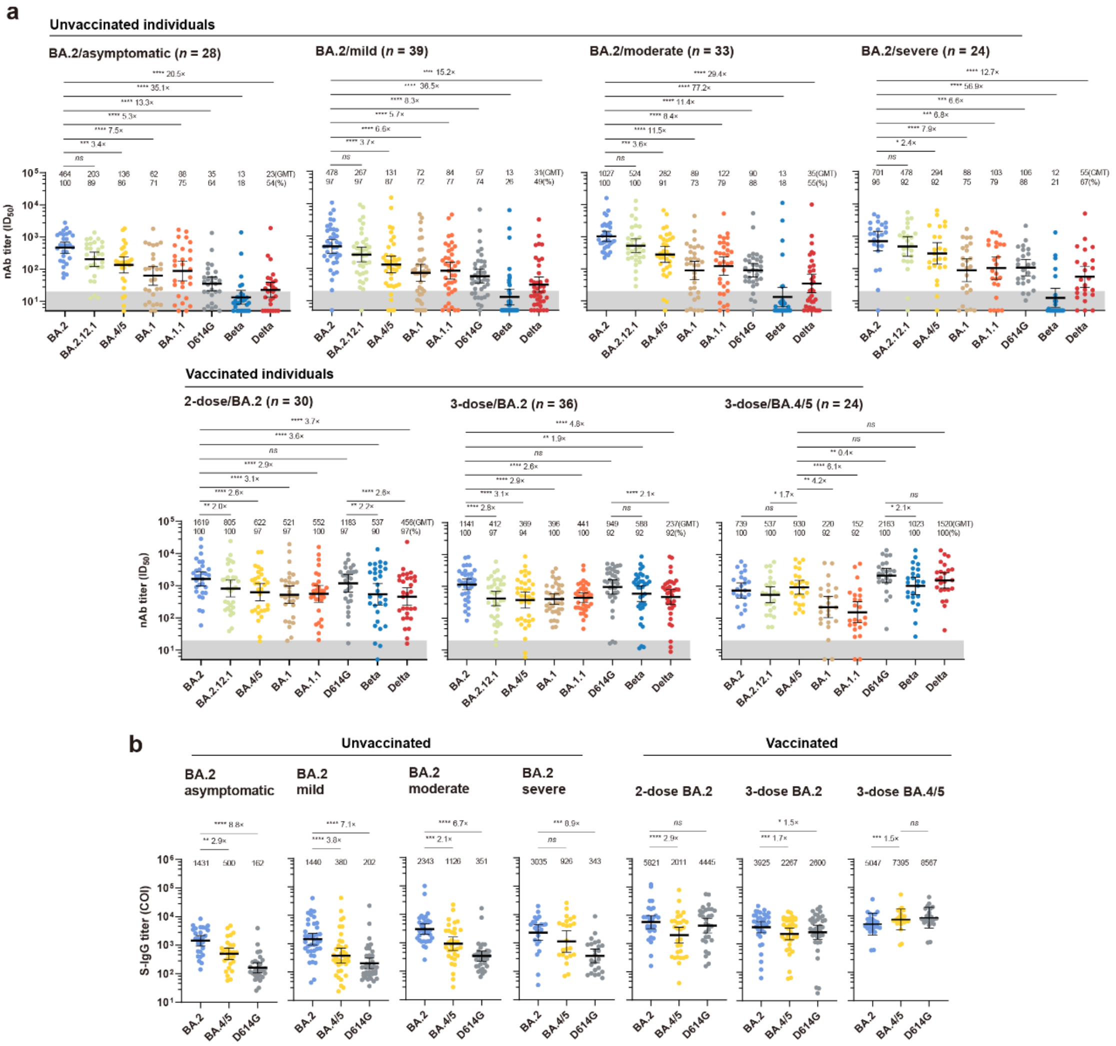

Supplementary Fig. S1 | Antibody response profiles of BA.2 or BA.4/5-infected patients.

a, Neutralization of D614G, Beta, Delta, and Omicron subvariants by HCPs from 7 groups of people with past BA.2 or BA.4/5 infections. Unvaccinated patients were divided by their disease severity during the acute phase of SARS-CoV-2 infection, including asymptomatic (*n* = 28), mild (*n* = 39), moderate (*n* = 33), and severe (*n* = 24). Breakthrough infected patients included 2- (*n* = 30) and 3-dose (*n* = 36) inactivated vaccine BA.2 breakthrough infected patients, and 3-dose inactivated vaccine BA.4/5 breakthrough infected patients (*n* = 24). **b,** S-IgG titer of BA.2, BA.4/5, and D614G by HCPs of various groups. Dark shadows indicate the limits of detections (LODs, ID_{50}=20). P-values were calculated using two-tailed Wilcoxon signed-rank tests of paired samples. *, *P*<0.05. **, *P*<0.01. ***, *P*<0.001. ****, *P*<0.0001. ns, not significant. nAb, neutralizing antibody. ID_{50}, half-maximal inhibitory dilution. COI, cutoff index. Data were plotted as the geometric mean with 95% confidence intervals (CI). The percentage in nAb titers plots indicated the percentage of samples above the detection limit.

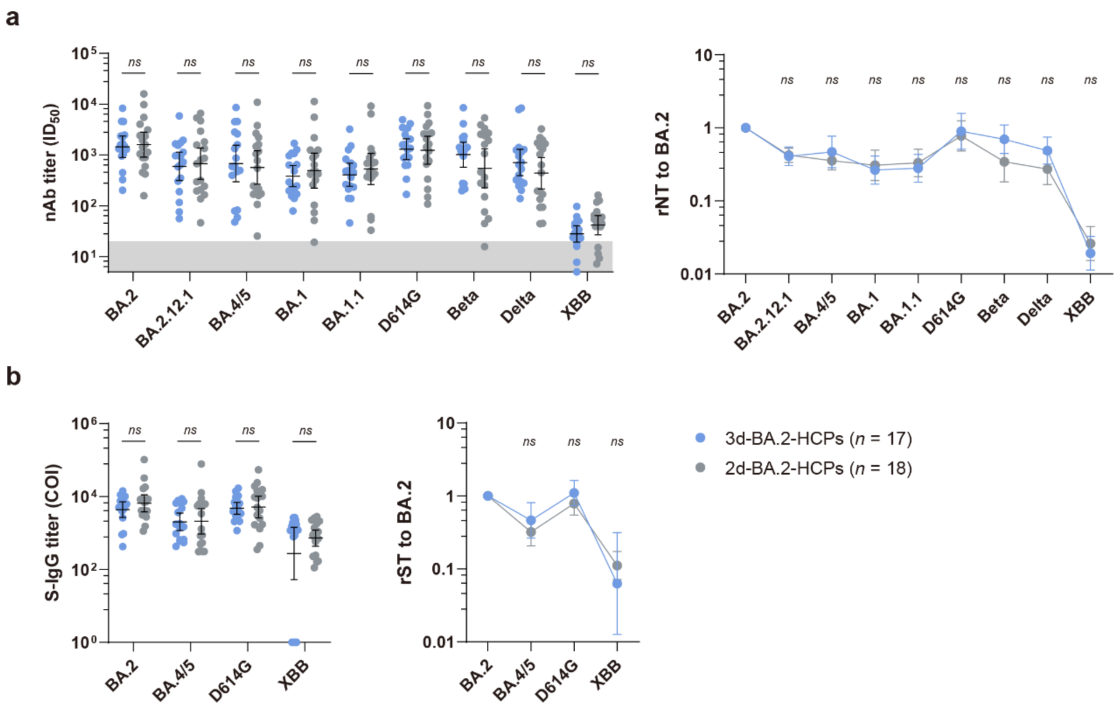

Supplementary Fig. S2 | Comparisons of nAb and S-IgG titers against spike variants between HCPs from 2- or 3-dose vaccinated individuals with BA.2 breakthrough infections.

Neutralization (**a**) and S-IgG (**b**) profiles of 2- (*n* = 18, 2d-BA.2-HCPs) or 3-dose (*n* = 17, 3d-BA.2-HCPs) vaccinated individuals with BA.2 breakthrough infections. Dark shadows indicate the LODs (ID_{50}=20). The rNT was calculated as its ID_{50} ratio against a variant to the BA.2 for each sample. The rST was calculated as its COI ratio against a variant to the BA.2 for each sample. P-values were calculated using two-tailed Wilcoxon signed-rank tests of paired samples. ns, not significant. nAb, neutralizing antibody. ID_{50}, half-maximal inhibitory dilution. COI, cutoff index. Data were plotted as the geometric mean with 95% CI.

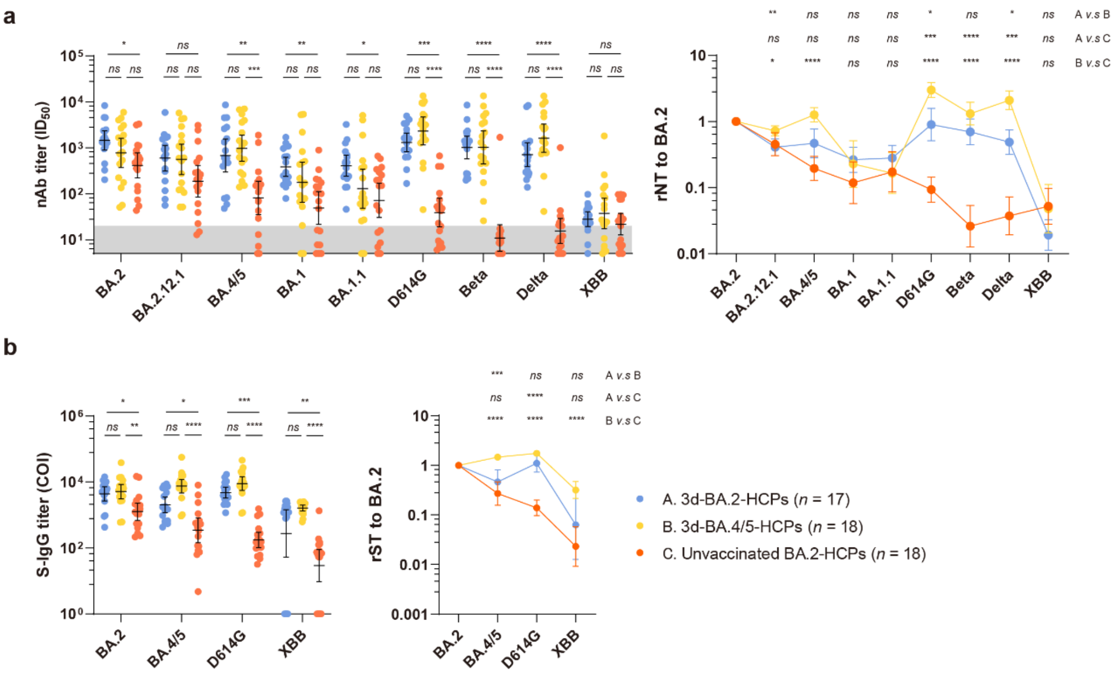

Supplementary Fig. S3 | Antibody response profiles of matched patients.

Neutralization (**a**) and S-IgG (**b**) profiles of HCPs from 3 matched clinical groups consisting of vaccinated individuals, including 3-dose (*n* = 17, 3d-BA.2-HCPs) inactivated vaccine BA.2 breakthrough infected patients, 3-dose inactivated vaccine BA.4/5 breakthrough infected patients (*n* = 18, 3d-BA.4/5-HCPs), and unvaccinated BA.2 infected patients (*n* = 18, Unvaccinated-BA.2-HCPs). The rNT was calculated as its ID_{50} ratio against a variant to the BA.2 for each sample. The rST was calculated as its COI ratio against a variant to the BA.2 for each sample. Dark shadows indicate the LODs (ID_{50}=20). P-values were calculated using two-tailed Wilcoxon signed-rank tests of paired samples. *, P<0.05. **, P<0.01. ***, P<0.001. ****, P<0.0001. ns, not significant. nAb, neutralizing antibody. ID_{50}, half-maximal inhibitory dilution. COI, cutoff index. Data were plotted as the geometric mean with 95% CI.

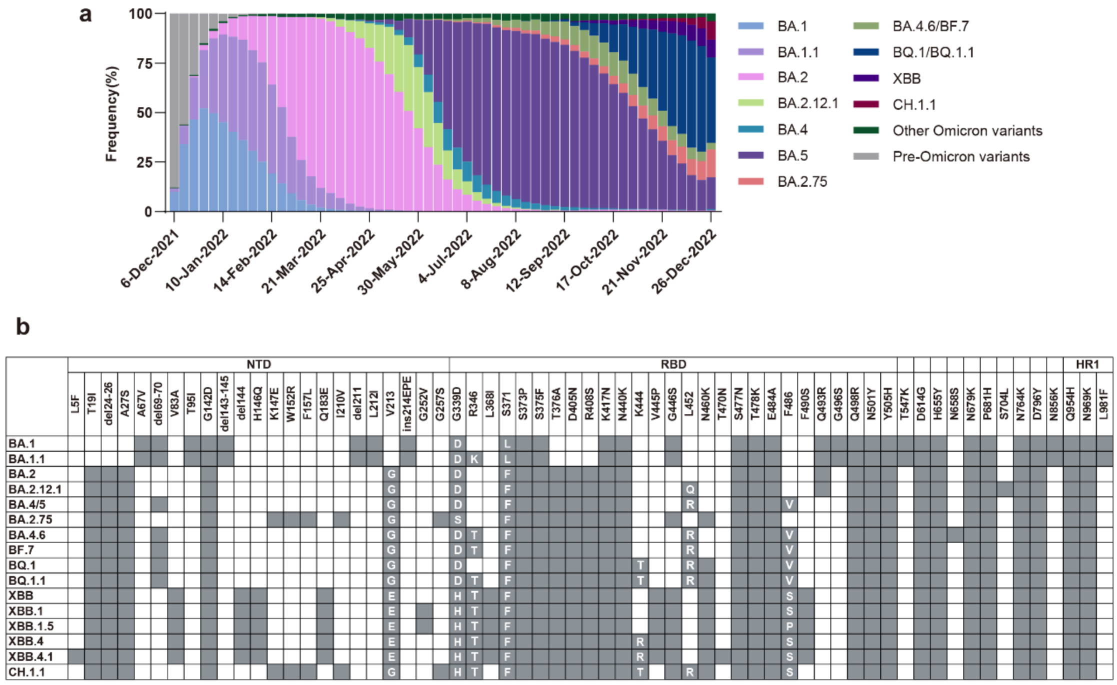

Supplementary Fig. S4 | Prevalence of SARS-CoV-2 Omicron subvariants.

a, Prevalence of SARS-CoV-2 variants around the world. **b,** Key spike mutations found in main Omicron subvariants. NTD, N-terminal domain. RBD, receptor binding site. HR, heptad repeat. del, deletion.

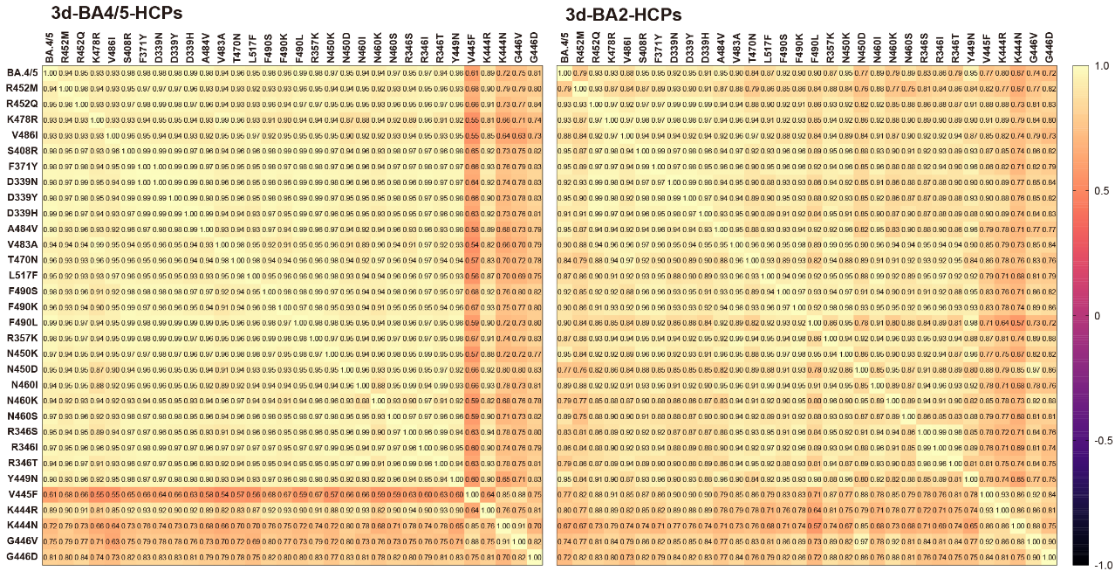

Supplementary Fig. S5 | Correlation of nAb titers to BA.4/5 and 34 BA.4/5-RBD mutants in breakthrough infected patients with 3-dose.

Data were presented as the Spearman correlation.

In BA.4/5 and its descendants

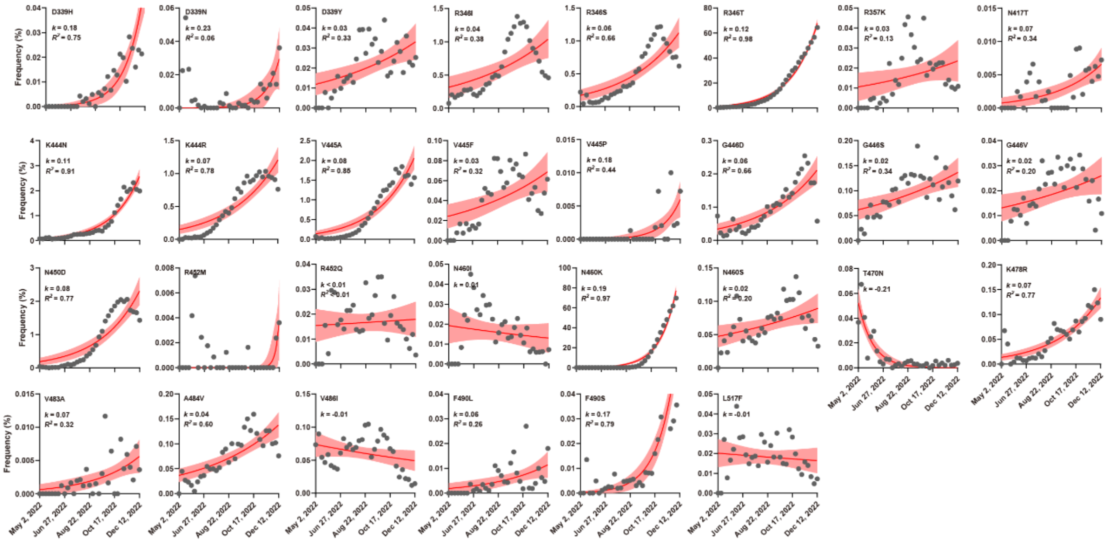

In BA.2 and its descendants

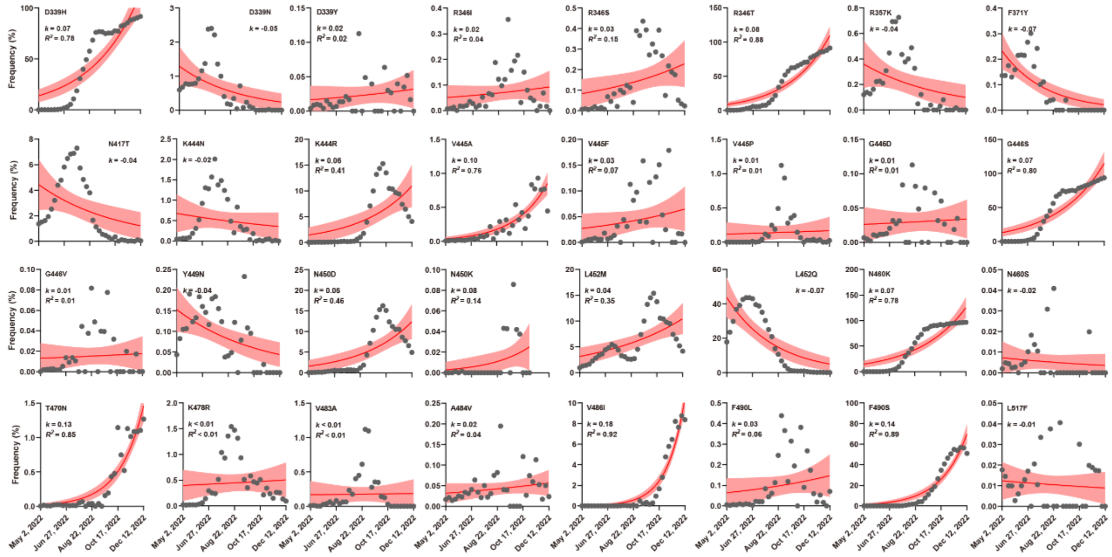

Supplementary Fig. S6 | Fitting results for the exponential growth in prevalence of selected mutations.

Frequency indicates the proportion of BA.4/5 or BA.2 and their sublines carrying the studied mutation among all strains. Because the number of sequences containing F371Y, Y449N, N450K, and F490K in BA.4/5 and its sublines, and the sequences containing N460I and F490K in BA.2 and its sublines were all less than 10, the prevalence of these mutations was not fitted. Related to Figure 1d-e.

a

		rhuACE2	LY-CoV1404	85F7	BD55-5840	COV2-2130
	D614G	108	0.5	0.7	3.5	43
	Beta	64	0.5	1.1	2.1	25
	Delta	29	0.4	1.1	1.3	80
	BA.1	82	0.7	6000	26	1183
	BA.1.1	39	0.5	6000	8.8	1011
	BA.2	60	0.5	14	48	56
	BA.2.12.1	27	0.4	8.1	14	47
	BA.4/5	59	0.4	5.8	14	195
	BQ.1	43	444	6000	57	6000
	BQ.1.1	64	4503	6000	2452	6000
	XBB	101	6000	6000	6000	6000
BA.4/5+	D339H	78	0.4	8.2	151	126
	D339N	42	0.4	11	141	144
	D339Y	76	0.4	10	511	104
	R346I	73	0.5	33	6000	817
	R346S	63	0.6	95	935	812
	R346T	54	0.5	58	2289	767
	R357K	89	0.5	18	21	196
	F371Y	82	0.4	16	14	113
	K417T	41	0.4	1.7	9.7	146
	K444N	150	1665	6000	282	6000
	K444R	82	1.0	73	14	1380
	V445A	106	11	9.4	8.0	6000
	V445F	81	47	11	14	4843
	V445P	52	6000	6000	850	6000
	G446D	130	2.1	6000	53	2138
	G446S	51	0.6	6000	18	1135
	G446V	148	155	6000	360	278
	Y449N	186	0.5	15	33	446
	N450D	105	0.5	6000	309	1069
	N450K	73	0.8	85	11	1003
	R452M	66	0.4	10	18	30
	R452Q	94	0.4	8.8	26	43
	N460I	41	0.4	7.9	10	105
	N460S	112	0.3	10	20	308
	N460K	119	0.3	10	54	204
	T470N	119	0.3	9.1	13	125
	K478R	57	0.3	7.4	8.5	98
	V483A	40	0.3	8.6	11	107
	A484V	93	0.5	8.6	76	229
	V486I	49	0.5	12	17	148
	F490L	184	0.4	11	25	563
	F490K	169	0.7	21	25	1030
	F490S	153	0.5	8.9	15	445
	L517F	137	0.3	8.7	10	110

neutralizing potency (IC_{50},ng/mL): <100 | 100~1000 | >1000

b

		rhuACE2	LY-CoV1404	85F7	BD55-5840	COV2-2130
	BQ.1	1.4	-1265	-1038	-4.2	-31
	BQ.1.1	-1.1	-12815	-1038	-178	-31
	XBB	-1.7	-17075	-1038	-436	-31
BA.4/5+	D339H	-1.3	-1.1	-1.4	-11	1.5
	D339N	1.4	-1.1	-1.9	-10	1.4
	D339Y	-1.3	-1.0	-1.8	-37	1.9
	R346I	-1.2	-1.5	-5.8	-436	-4.2
	R346S	-1.1	-1.6	-16	-68	-4.2
	R346T	1.1	-1.4	-10	-167	-3.9
	R357K	-1.5	-1.5	-3.2	-1.5	-1.0
	F371Y	-1.4	-1.2	-2.8	-1.0	1.7
	K417T	1.4	-1.0	3.4	1.4	1.3
	K444N	-2.5	-4738	-1038	-21	-31
	K444R	-1.4	-2.9	-13	-1.0	-7.1
	V445A	-1.8	-32	-1.6	1.7	-31
	V445F	-1.4	-135	-1.9	-1.0	-25
	V445P	1.1	-17075	-1038	-62	-31
	G446D	-2.2	-5.9	-1038	-3.9	-11
	G446S	1.1	-1.8	-1038	-1.3	-5.8
	G446V	-2.5	-442	-1038	-26	-1.4
	Y449N	-3.1	-1.4	-2.6	-2.4	-2.3
	N450D	-1.8	-1.5	-1038	-23	-5.5
	N450K	-1.2	-2.2	-15	1.2	-5.2
	R452M	-1.1	-1.0	-1.7	-1.3	6.5
	R452Q	-1.6	-1.1	-1.5	-1.9	4.5
	N460I	1.4	-1.1	-1.4	1.3	1.8
	N460S	-1.9	1.0	-1.8	-1.5	-1.6
	N460K	-2.0	1.0	-1.7	-3.9	-1.0
	T470N	-2.0	1.1	-1.6	1.1	1.6
	K478R	1.0	1.2	-1.3	1.6	2.0
	V483A	1.5	1.1	-1.5	1.3	1.8
	A484V	-1.6	-1.4	-1.5	-5.5	-1.2
	V486I	1.2	-1.3	-2.0	-1.2	1.3
	F490L	-3.1	-1.1	-2.0	-1.8	-2.9
	F490K	-2.9	-1.9	-3.7	-1.8	-5.3
	F490S	-2.6	-1.3	-1.5	-1.1	-2.3
	L517F	-2.3	1.1	-1.5	1.4	1.8

relative potency: >3 | 3~-3 | -3~-10 | -10~-100 | over -100×

c

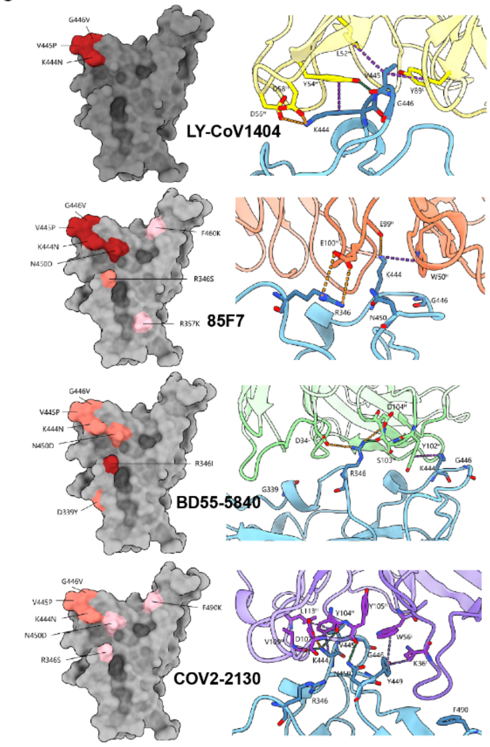

d

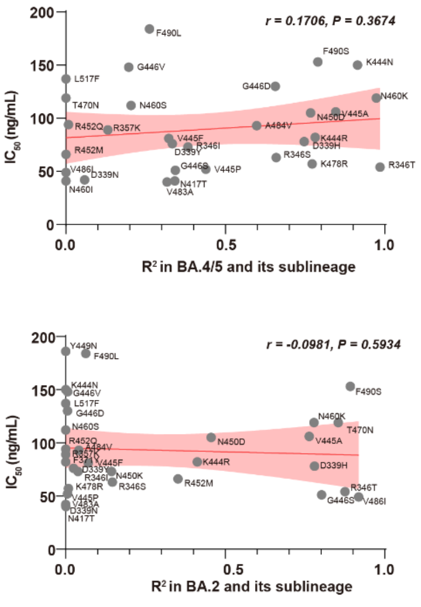

Supplementary Fig. S7 | Influence of additional RBD mutations in the context of BA.4/5 spike on activities of broadly neutralizing antibodies and rhuACE2.

a, IC_{50} fold changes relative to BA.4/5 of mAbs and rhuACE2 in neutralizing various BA.4/5-RBD mutants. Data were derived from the mean value of ≥3 technical replicates. The

number marked with a negative sign signifies the multiple of descent. **b,** IC50 (at ng/mL) of mAbs and rhuACE2 against the variants 34 BA.4/5-RBD mutants and other SARS-CoV-2 variants. **c,** Positions of mutations affecting each mAb in RBD and interactions between RBD and LY-CoV1404, 85F7, BD55-5840, and COV2-2130. The colors on the left indicate the magnitude of the neutralization effect on mAbs, and the color scales are the same as in panel (a). Hydrophobic interaction, hydrogen bonds, and salt bridge are marked as purple, green, and yellow-red dashed lines, respectively. **d,** Correlation of IC_{50} of rhuACE2 against the 34 BA.4/5-RBD mutants, with the R^2 of exponential growth fit of the prevalence of point mutations in BA.4/5 or and their sublines. P-values resulted from a two-tailed test for the Spearman rank correlation coefficient.

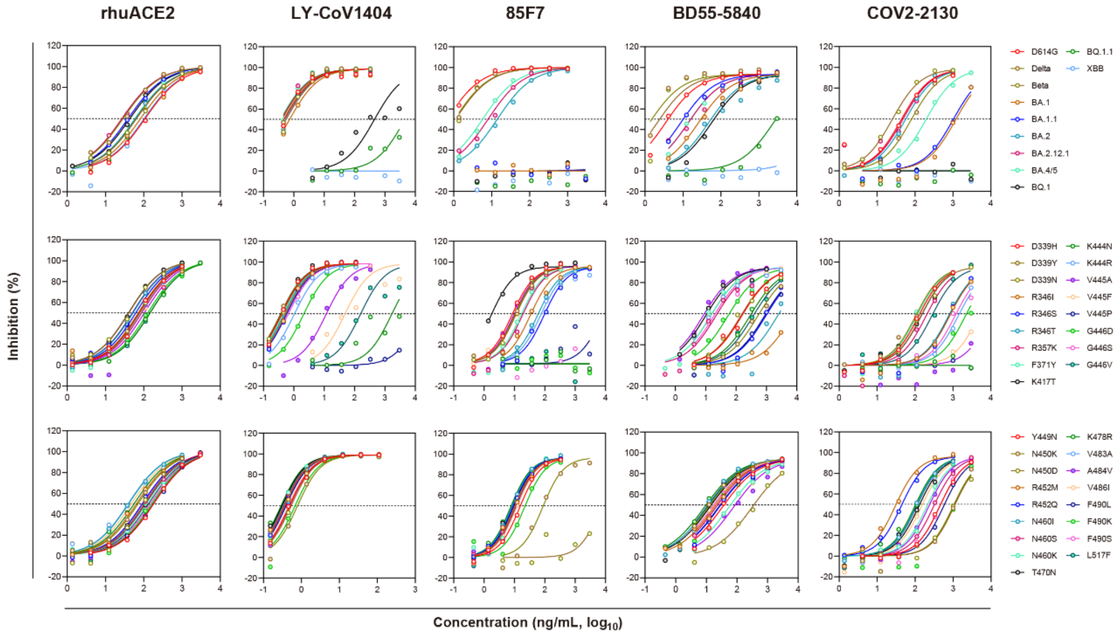

Supplementary Fig. S8 | Neutralization profiles for mAbs and rhuACE2 against 11 variants and 34 BA.4/5-RBD mutants.

All mAbs were tested at 3-fold serial dilutions, related to Supplementary Figure S7a-b. The data were plotted as the mean value of ≥3 technical replicates.

Supplementary Tables

Table S1 | Characteristics of all the participants.

Characteristics	BA.2 asymptomatic	BA.2 mild	BA.2 moderate	BA.2 severe	3-dose BA.2	2-dose BA.2	3-dose BA.4/5
**N**	28	39	33	24	36	30	24
**Median age (yr, IQR)**	64 (58-73)	77 (68-88)	83 (67-87)	84 (80-88)	48 (37-56)	48 (34-59)	43 (24-52)
**Age group (*n*, %)**							
<18 yr	0 (0)	1 (2.6)	0 (0)	0 (0)	0 (0)	2 (6.7)	0 (0)
18~64 yr	14 (50.0)	8 (20.5)	5 (15.2)	0 (0)	32 (88.9)	24 (80.0)	24 (100)
>=65 yr	14 (50.0)	30 (76.9)	28 (84.8)	24 (100)	4 (11.1)	4 (13.3)	0 (0)
**Male (*n*, %)**	16 (57.1)	20 (51.3)	14 (42.4)	10 (41.7)	14 (38.9)	15 (50.0)	14 (58.3)
**Co-existing medical conditions (*n*, %)**	16 (57.1)	20 (51.3)	23 (69.7)	17 (70.8)	6 (16.7)	9 (30.0)	6 (25.0)
**Illness (*n*, %)**							
Asymptomatic	-	-	-	-	21 (58.3)	16 (53.3)	0 (0)
Mild	-	-	-	-	15 (41.7)	11 (36.7)	8 (33.3)
Moderate	-	-	-	-	0 (0)	3 (10.0)	16 (66.7)
Severe	-	-	-	-	0 (0)	0 (0)	0 (0)
**Sampling after first qPCR positive (days, IQR)**	34 (18-92)	24 (16-90)	23 (16-35)	29 (18-38)	31 (16-90)	27 (16-100)	9 (9-12)

yr, years. IQR, interquartile range.

Table S2 | Characteristics of matched participants.

Characteristics	3d-BA.2-HCPs (1)	3d-BA.4/5-HCPs (2)	Unvaccinated BA.2-HCPs (3)	2d-BA.2-HCPs (4)	(1) vs (2)	(1) vs (3)	(3) vs (2)	(1) vs (4)
**N**	17	18	18	18				
**Median age (yr, IQR)**	54 (46-59)	48 (26-55)	52 (34-62)	46 (35-56)	0.083	0.620	0.100	0.095
**Age group (*n*, %)**								
<18 yr	0 (0)	0 (0)	0 (0)	0 (0)				
18~64 yr	14 (82.4)	18 (100)	16 (88.9)	16 (88.9)	0.104	0.658	0.486	0.658
>=65 yr	3 (17.6)	0 (0)	2 (11.1)	2 (11.1)				
**Male (*n*, %)**	5 (29.4)	9 (50.0)	11 (61.1)	9 (50.0)	0.370	0.123	0.737	0.370
**Co-existing medical conditions (*n*, %)**	4 (23.5)	5 (27.8)	9 (50.0)	7 (38.9)	>0.999	0.164	0.305	0.471
**Illness (*n*, %)**								
Asymptomatic	11 (64.7)	0 (0)	10 (55.6)	11 (61.1)				
Mild	6 (35.3)	4 (22.2)	5 (27.8)	5 (27.8)	<0.001	0.337	<0.001	0.558
Moderate	0 (0)	14 (77.8)	3 (16.7)	2 (11.1)				
Severe	0 (0)	0 (0)	0 (0)	0 (0)				
**Sampling after first qPCR positive (days, IQR)**	16 (13-17)	10 (9-13)	37 (18-91)	26 (17-98)	0.001	<0.001	<0.001	<0.001

The Chi-Square statistic was used for categorical variables. Continuous variables were compared by using a two-sided Mann-Whitney U-test. Group (1) and (2) were involved in antigenic analyses of the BA.4/5-RBD mutants. yr, years. IQR, interquartile range.
